# P-651. An Artiﬁcial Intelligence Model Outperformed Experienced Clinicians in Differentiating the Aetiology of Pneumonia on Chest Computed Tomography: A Retrospective Study

**DOI:** 10.1093/ofid/ofae631.848

**Published:** 2025-01-29

**Authors:** Wenting Jin, Ying Shao, Yaozong Gao, Bijie Hu

**Affiliations:** Zhongshan Hospital, Fudan University, Shanghai, Shanghai, China (People's Republic); Shanghai United Imaging Intelligence Co., Ltd., Shanghai, Shanghai, China; Shanghai United Imaging Intelligence Co., Ltd., Shanghai, Shanghai, China; Department of Infectious Diseases, Zhongshan Hospital, Fudan University, Shanghai, Shanghai, China

## Abstract

**Background:**

Rapid and precise aetiological diagnosis is crucial for managing pneumonia. We aimed to develop and validate deep learning (DL) models for differentiating ten pneumonia aetiologies on chest computed tomography images.

The area under the receiver operating characteristic curve (ROC) and the area under the curve (AUC) for the two deep learning models, the performance of four different radiologists and four different pulmonologists (A-B); The ROC and AUC of two models in identifying 10 different classifications (C-F).

**Figure:**
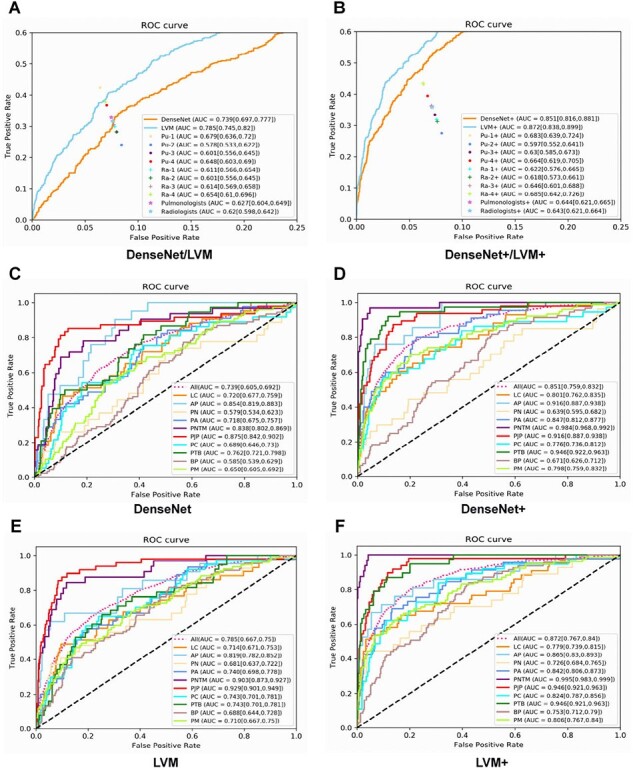

Abbreviation: BP, bacterial pneumonia; AP, atypical pneumonia; PN, pulmonary nocardiosis; PTB, pulmonary tuberculosis; PNTM, pulmonary nontuberculous mycobacterial infection; PC, pulmonary cryptococosis; PA, pulmonary aspergillosis; PJP, pneumocystis jirovecii pneumonia, LC, lung cancer; PM, pneumonia mimics; Ra, radiologists; Pu, pulmonologists.

**Methods:**

We enrolled 1091 pneumonia patients with one of 10 definite aetiological diagnoses from Zhongshan Hospital between Oct. 1^st^, 2015, and Jun. 30^th^, 2022, in this retrospective observational study. We trained and validated two DL models: a classic 3D-DenseNet model and a novel large vision model (LVM). The models were tested on a data from 59 nonoverlapping patients for external dataset. Model performance was assessed using the area under the curve (AUC) of the Top1 diagnosis and the accuracy of the Top1, Top2, and Top3 diagnoses. Comparisons were also performed between the DL models and eight experienced radiologists and pulmonologists.

The accuracy of Top1, Top2 and Top3 diagnoses of two deep learning models, radiologists and pulmonologists with or without nonimaging data

**Figure:**
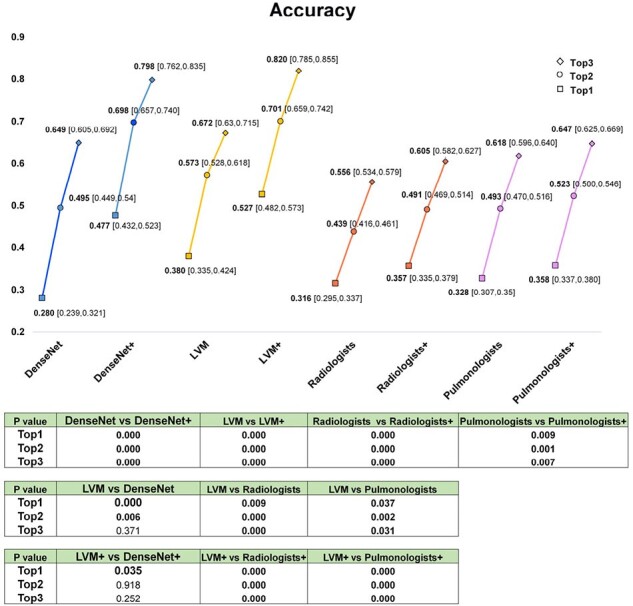

Abbreviation: LVM, large vision model; Note: + means with nonimaging data

**Results:**

The LVM+ had a greater average prediction performance than DenseNet+, radiologists+ and pulmonologists+, with Top1 AUCs of 0.872, 0.851, 0.643 and 0.644, respectively. The Top1, Top2, and Top3 accuracies of LVM+ were 0.527, 0.701 and 0.820, respectively, similarly outperforming DenseNet+, radiologists+ and pulmonologists+. The two models performed similarly in the external test sets, with AUCs of 0.775 for LVM and 0.743 for DenseNet. The classification-related confusion matrix of LVM/DenseNet showed a significant advantage in identifying PNTM and PJP.

**Conclusion:**

This study represents the most complete classification that best matches the diagnosis of pneumonia in realistic clinical settings. We expect this method to be applied clinically to foster novel approaches to improve the accuracy in diagnosing pneumonia.

**Disclosures:**

**All Authors**: No reported disclosures

